# Allergic diseases of the skin and drug allergies – 2028. Vitamin D insufficiency in dress syndrome

**DOI:** 10.1186/1939-4551-6-S1-P114

**Published:** 2013-04-23

**Authors:** Young-Hee Nam, Il-Hwan Jeong, Hye-Won Lee, Neul-Bom Yoon, Soo-Jung Um, Choon-Hee Son, Soo-Keol Lee

**Affiliations:** 1 Department of Internal Medicine, College of Medicine, Dong-A University, Busan, South Korea

## Background

Vitamin D deficiency (VDD) is widespread and on the increase. A few reports showed VDD has been implicated cutaneous symptoms such as rash and urticaria/angioedema and drug reaction with eosinoophilia and systemic symptoms (DRESS). We evaluated the association of serum 25-hydroxyvitamin D3(25[OH]D3) and DRESS.

## Methods

36 patients diagnosed as DRESS were prospectively collected from September 2010 to April 2012. The diagnostic criteria in this study was used from our previous report.

## Results

Study patients consisted of 16 men (44.4%) and 13 women (55.6%). The most common causative drugs were antibiotics (17, 47.2%) and anticonvulsants (9, 25%), followed by non-steroidal anti-inflammatory drugs (5, 13.2%), antituberculosis drugs (4, 11.1%), undetermined agents (4, 8.9%), others (2, 5.6%) and undetermined (2, 5.6%). The mean serum 25[OH]D3 level of the total subjects was 11.96 ± 10.27 ng/ml. Thirty-five patients (97.2%) had low vitamin D levels; 19 were severe VDD (<10 ng/mL, group±), and 16 vitamin D insufficiency (10-30 ng/mL, groupII). The mean serum 25[OH]D3 level of each group was 7.02 ± 1.65 ng/ml and 14.46 ± 3.56 ng/ml, respectively.There were no significant differences in sex, age, culprit drugs, organ involvements and the use of systemic steroids between two groups, except admission days (96.21 ± 89.66 vs. 37.56± 40.43, p=0.034). The level of serum 25(OH)D3 was inversely correlated with admission days (r=-0.387, p=0.02).

## Conclusions

Vitamin D insufficiency was noted in patients with DRESS. Further studies are needed in large samples and to evaluate the vitamin D roles in drug hypersensitivity.

